# Number of rare germline CNVs and *TP53* mutation types

**DOI:** 10.1186/1750-1172-7-101

**Published:** 2012-12-21

**Authors:** Amanda G Silva, Isabel Maria W Achatz, Ana CV Krepischi, Peter L Pearson, Carla Rosenberg

**Affiliations:** 1 International Center for Research and Training, A. C. Camargo Hospital, Rua Taguá, 440, 01508-010, São Paulo, Brazil; 2 Department of Genetics and Evolutionary Biology, Institute of Biosciences, University of São Paulo, Rua do Matão, 277, 05422-970, São Paulo, Brazil

**Keywords:** *TP53*, R337H, Li-Fraumeni syndrome, CNV, Penetrance

## Abstract

**Background:**

The Li-Fraumeni syndrome (LFS), an inherited rare cancer predisposition syndrome characterized by a variety of early-onset tumors, is caused by different highly penetrant germline mutations in the *TP53* gene; each separate mutation has dissimilar functional and phenotypic effects, which partially clarifies the reported heterogeneity between LFS families. Increases in copy number variation (CNV) have been reported in *TP53* mutated individuals, and are also postulated to contribute to LFS phenotypic variability. The Brazilian p.R337H *TP53* mutation has particular functional and regulatory properties that differ from most other common LFS *TP53* mutations, by conferring a strikingly milder phenotype.

**Methods:**

We compared the CNV profiles of controls, and LFS individuals carrying either p.R337H or DNA binding domain (DBD) *TP53* mutations by high resolution array-CGH.

**Results:**

Although we did not find any significant difference in the frequency of CNVs between LFS patients and controls, our data indicated an increased proportion of rare CNVs per genome in patients carrying DBD mutations compared to both controls (p=0.0002***) and p.R337H (0.0156*) mutants.

**Conclusions:**

The larger accumulation of rare CNVs in DBD mutants may contribute to the reported anticipation and severity of the syndrome; likewise the fact that p.R337H individuals do not present the same magnitude of rare CNV accumulation may also explain the maintenance of this mutation at relatively high frequency in some populations.

## Background

Li-Fraumeni syndrome (LFS; OMIM #151623) is an autosomal dominant highly penetrant cancer predisposition syndrome characterized by a variety of early onset tumors [1,2]. LFS arises from germline mutations in the *TP53* gene, which codes for a transcription factor implicated in cell proliferation, apoptosis, and genomic stability [3,4]. Although decreases in the age of cancer onset and increases in cancer incidence over successive generations have been documented in LFS [5,6], the molecular basis of this anticipation is still a matter of debate.

Most mutations in the *TP53* gene affect the DNA binding domain (DBD) and result in missense substitutions giving rise to altered proteins [7] that have a considerable longer half-life than the wild-type protein, resulting in accumulation of the mutant protein, at least in transfected or neoplastic cells [8].

It is known that different mutant p53 proteins may have diverse functional and biological effects, which could partially explain the heterogeneity reported between Li-Fraumeni families [9]. Not only the type of *TP53* mutation determines the tumor spectrum and severity of phenotype, but other germline genetic factors such as polymorphisms for *TP53* and *MDM2*[10] and telomere length [11,12] are assumed to modulate the cancer phenotype. It has been more recently observed that individuals carrying *TP53* mutations show a ~3-fold increase in DNA copy number variation (CNV) compared to controls, suggesting that CNVs make an additive contribution to cancer risk [13].

In Brazil, the Li-Fraumeni syndrome also occurs as a frequent cancer predisposition alteration due to a germline mutation at codon 337 (c.1010G4A, p.R337H) that has been identified in families matching LFS definitions [14,15]. The frequency of p.R337H in the population of Southern Brazil is about 1:280 individuals, about 300 times higher than any other single germline *TP53* mutation [16]. Analysis of 28 polymorphic markers showed p.R337H to have arisen from a founder mutation that has been spreading in the Brazilian population since the XVIII century [17,18]. This mildly pathogenic mutation shows a penetrance for cancer of less than 20% by age 30, compared to about 50% in classic LFS [18]: this provides more opportunity for transmitting the mutation to the next generations, which explains, at least in part, the maintenance in the Brazilian population.

Structural and functional studies have demonstrated that the p.R337H protein displays a pH-dependence, rendering the protein inactive only under conditions of increased intracellular pH, and the majority of the time behaves normally, which could explain its lower overall malignant potential [19].

It is accepted that differences in severity and age of onset of cancer manifestation linked to various germline *TP53* mutations not only result from the *TP53* mutations themselves, but also from their interaction with other genetic variants, of which CNVs are now becoming recognized as a significant contributant (reviewed in [20]). Based on the foregoing considerations we hypothesize that different types of *TP53* mutations exhibit different CNV profiles reflecting the genotype- phenotype correlation and also possibly playing a role in the anticipation observed in LFS families.

## Methods

### Patients

The patients were seen in the Department of Oncogenetics in the A. C. Camargo Hospital, São Paulo, Brazil. Research protocol approval was provided by the ethics committee of the institution, and informed consent obtained from all subjects and their families. The cohort comprised 21 probands with *TP53* germline mutations [14]. The characteristics of the mutations, cancer type, age at onset and clinical classification of the patients are described in Table 1.

**Table 1:** Clinical data of the patients and characteristics of the detected mutations

**ID**	**Classification**	**Gender**	***TP53 *mutation**	**Effect**	**p53 protein domain function**	**Tumor type (age)**
Y1T0	Chompret	F	R213Q CGA>CAA	Missense	DNA binding	Hydathiform mole (23), Ampulla of Vater (41)
Y33T0	LFL Birch	F	V173M GTG>ATG	Missense	DNA binding	Soft tissue sarcoma (23), Breast (43)
Y53T0	Eeles1	F	G245S GGC>AGC	Missense	DNA binding	Bilateral breast (36), Gall bladder (?)
Y57T0	Eeles1	F	G244D GGC>GAC	Missense	DNA binding	Breast (40), Colorectal (?)
Y65T0	Chompret	F	V197M GTG >ATG	Missense	DNA binding	Colorectal (45), Lung (51), Soft tissue sarcoma (51)
Y79T0	Chompret	F	T125T ACG>ACA	Splice	DNA binding	Adrenal carcinoma (1)
Y87T0	LFL/Chompret	M	S241Y TCC>TAC	Missense	DNA binding	Rhabdomyosarcoma (2), Choroid plexus tumor (7), Liposarcoma (10)
Y97T1	LFS	F	IVS8+1G>A	Splice	DNA binding	Breast (27)
Y103T2	LFL/Chompret	F	H214Q	Missense	DNA binding	Breast (62)
Y12T1	LFS	F	R337H CGC>CAC	Missense	Tetramerisation	Soft tissue sarcoma (58), Breast (59), Thyroid (61), Soft tissue sarcoma (62)
Y15T0	Chompret	F	R337H CGC>CAC	Missense	Tetramerisation	Adrenal carcinoma (6), Kidney (7)
Y27T0	LFL Birch	F	R337H CGC>CAC	Missense	Tetramerisation	Breast (36)
Y49T1	Eeles 1	M	R337H CGC>CAC	Missense	Tetramerisation	Kidney (64)
Y99T0	Chompret	F	R337H CGC>CAC	Missense	Tetramerisation	Breast (32)
Y100T0	LFS	F	R337H CGC>CAC	Missense	Tetramerisation	Breast (46)
Y106T0	Chompret	F	R337H CGC>CAC	Missense	Tetramerisation	Breast (43)
Y107T0	LFS	M	R337H CGC>CAC	Missense	Tetramerisation	Adrenal carcinoma (2)
Y127T0	Chompret	M	R337H CGC>CAC	Missense	Tetramerisation	Adrenal carcinoma (3)
Y131T0	LFS	F	R337H CGC>CAC	Missense	Tetramerisation	Leg synovial sarcoma (32)
Y144T0	Chompret	F	R337H CGC>CAC	Missense	Tetramerisation	Breast (29)
Y154T0	Chompret	F	R337H CGC>CAC	Missense	Tetramerisation	Breast (48)

### Controls

The CNV control data used in this study was obtained from a previously reported group of 100 random individuals from the urban area of São Paulo, Brazil [21].

### Array-CGH

We performed oligonucleotide microarray-based CGH (array-CGH) using a 4 × 180 K whole-genome platform (design 22060, Agilent Technologies, Santa Clara, USA), which has an average spacing of 18 Kb between probes. Briefly, samples were labeled with Cy3- and Cy5-dCTPs by random priming; purification, hybridization, and washing were carried out as recommended by the manufacturer. Scanned images of the arrays were processed and analyzed using Feature Extraction software and Genomic Workbench software (both from Agilent Technologies), with the statistical algorithm ADM-2, and a sensitivity threshold of 6.7. We applied a′loop design′ in our hybridizations as previously described [22], resulting in two reverse labeling hybridizations per sample. We considered a gain or loss in copy number when the log_2_ ratio of the Cy3/Cy5 intensities of a given genomic segment was >0.5 or <−0.8, respectively. Alterations had to encompass at least three consecutive probes to be called by the software, and those not detected in both dye-swap experiments of the same sample were excluded from the analysis.

### Analysis

The detected copy number variations were compared to CNVs reported in the Database of Genomic Variants (DGV; http://projects.tcag.ca/variation/; freeze December, 2011). We classified the CNVs into ″rare″ and ″common″, considering as ″rare″ those CNVs where the imbalances encompassed coding sequences which were never or only once reported in DGV. Mann–Whitney and Fisher-exact tests were used to evaluate CNVs regarding proportions of total and rare CNVs, frequency of deletions and duplications and size of the variation.

The frequencies were then calculated for each rare variant in the total population described in the DGV studies. For estimating the total population in DGV, BAC array publications have been excluded because they are known to overestimate the CNV boundaries; the remaining 22 studies comprised a total of 8058 individuals investigated. The frequencies of these rare variants were than cross-checked with a study comprising 8148 controls [23], which is not documented in DGV but is registered in dbVar (http://www.ncbi.nlm.nih.gov/dbvar).

## Results

The CNV data of this study are summarized in Table 2; a full description of the CNVs identified in the patients can be found in Additional file 1. The overall frequency of CNVs per genome did not differ either between patients and controls or between p.R33H and DBD mutants (Mann–Whitney test). No significant differences were detected between the groups regarding CNV size or frequency of deletions and duplications.

**Table 2:** **Copy number variation data in controls and LFS/LFL patients classified according to *TP53 *mutation type**

	**Controls**	**p.R337H carriers**	**DBD mutation carriers**
**Number of individuals**	**100**	**12**	**9**
**Number of CNVs**	**702**	**92**	**66**
Common	679	89	56
Rare	23	3	10
**Average number of all CNVs per diploid genome**	**7.0±3.1**	**7.7±2.2**	**7.3±2.5**
**Average number of rare CNVs per diploid genome**	**0.2±0.4**	**0.2±0.6**	**1.1±1.3**^**a**^
**Proportion of rare/common CNVs**	**0.034**	**0.034**^**b**^	**0.179**^**c**^

a. difference between controls and DBD mutation carriers (p=0.0026**).

b. difference between p.R337H and DBD mutation carriers (p=0.0156*).

c. difference between controls and DBD mutation carriers (p=0.0002***).

However, with regards to rarity, we found 13 rare CNVs among all LFS patients, including 9 deletions and 4 duplications. Additional file 2 describes the rare CNVs**,** the studies in which they have been observed and their corresponding frequencies in the combined individuals of DGV and dbVar. The calculated frequencies of these rare variants in normal populations as estimated based both on DGV and dbVar were < 0.1%, a value much inferior to the 1% threshold often associated with polimorphisms.

**Figure 1:**
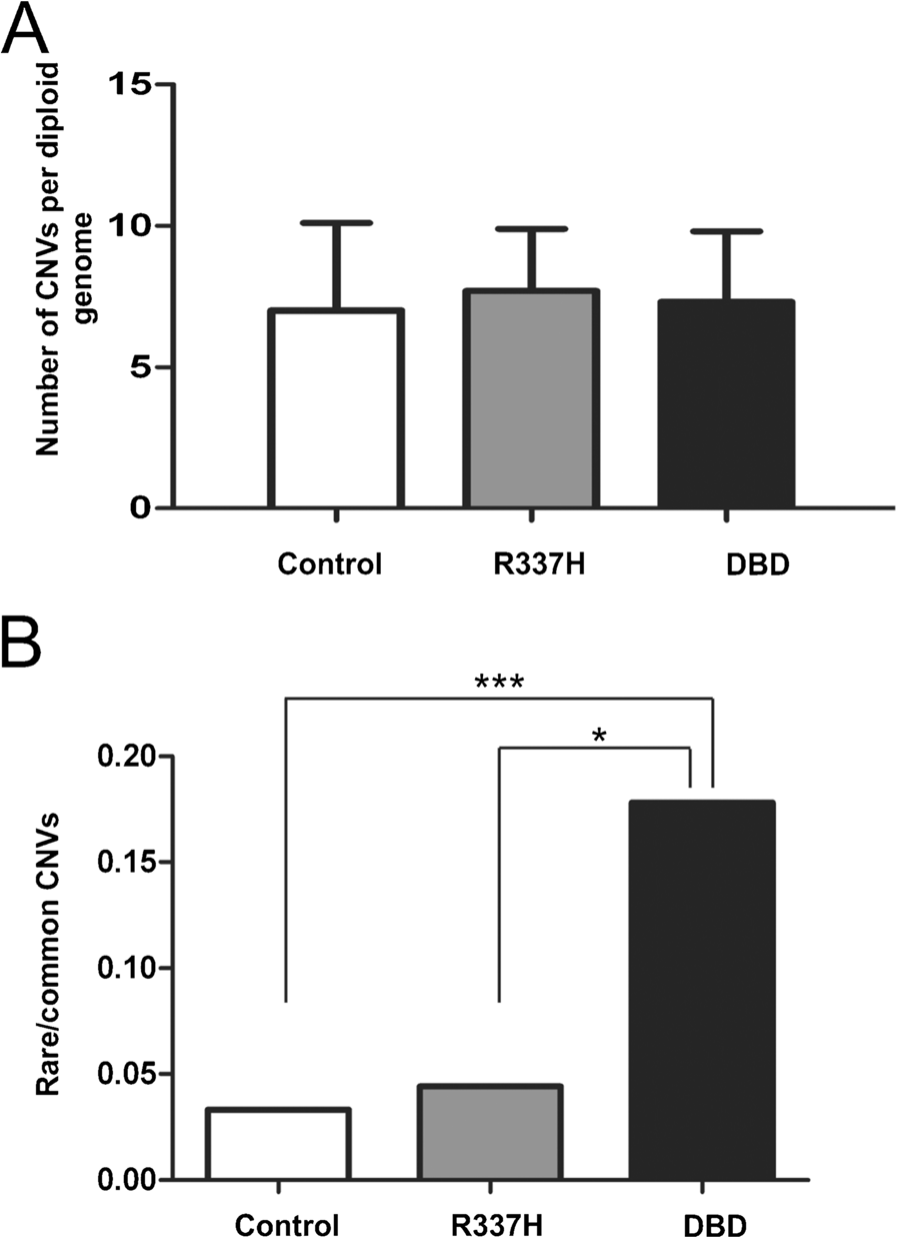
**Distribution of CNVs according to *TP53 *mutation status.** The graphs show no differences in the frequency of total CNVs but an increased frequency of rare CNVs in DBD mutation carriers. **(A)** Frequency of total CNVs in LFS patients and controls; **(B)** Rare/common CNV ratio in LFS patients and controls. White bar represents the control individuals, grey bar represents p.R337H mutated carriers and dark black bar the *TP53* DBD mutated carriers. Fisher-exact test; *p=0.0156 and ***p=0.0002.

In comparison to our control group, *TP53* mutated individuals (p.R337H and DBD) did not exhibit differences in the total number of CNVs/genome (Figure 1A). However, we did find a highly significant increase in the rare/common CNV ratio in the LFS patients carrying mutations in the *TP53* DBD when compared to both controls (p=0.0002) and to patients carrying the p.R337H mutation (p=0.0156; Fisher-exact test) (Figure 1B). Interestingly, no significant difference was found between controls and p.R337H patients.

## Discussion

The vast majority of *TP53* mutations lies within the DNA binding domain and target critical sites that either make contact with DNA or are required to maintain appropriate p53 protein conformation. Such alterations lead to a significant loss of *TP53* transcription and tumor suppressive activity, with great impact on genomic stability and apoptosis [24-26]. In contrast, the p.R337H mutation, which affects a residue of the oligomerization domain of the p53 protein, has a markedly less severe impact on tumor predisposition, which correlates with its remaining p53 activity [19,27].

Shlien et al. [13] reported a ~3-fold increase in the frequency of CNVs in *TP53* germline mutation carriers compared to normal individuals, and proposed that this increase might result from enhanced genome instability in the presence of *TP53* mutations. In our study, we found no difference between the total number of germline CNVs present in LFS patients and controls; however, we noted a highly significant increase (>5 fold) in the rare CNVs in *TP53* DBD mutation carriers as compared both to controls and to p.R337H carriers. A difference between Shlien et al. [13] data and ours refers to the controls samples: in their work data previously assembled in other studies were used to establish a baseline CNV control frequency, whereas our control and patient samples have been investigated in parallel in the same laboratory. We should also point out that the studies were carried out using different microarray technologies, namely array-CGH and SNP platforms. In fact, a large study comparing different platforms found that different analytic tools typically yield CNV calls with <50% concordance, overall regarding smaller CNVs [28].

An increase of only rare germline CNVs in the DBD *TP53* carriers, rather than an overall increase in CNVs, suggests that a selective mechanism would be involved in this event. Under the assumption that pathogenic variants would only remain in the population at low frequency, the selection against new pathogenic CNVs (in the context of studies on schizophrenia and autism, respectively) is extremely high [29,30]. The finding of an increased proportion of rare CNVs, potentially pathogenic, among *TP53* DBD mutants may be the result of an inefficient selection against new CNVs (deficient apoptosis). This hypothesis could be tested by determining the pattern of inheritance of the CNVs [13], but unfortunately parents were not available for investigation.

Anticipation, characterized by an increase of penetrance and earlier age of onset over subsequent generations, has been well documented for LFS [5,6]. An accumulation of germline CNVs impacting cancer predisposition in LFS provides a logical explanation for anticipation. How CNVs and other genetic modifiers interact and modulate *TP53* tumor suppressor activities remains to be determined. Elucidating these mechanisms may hold the key to defining evidence-based strategies for diagnosis, counseling, follow-up and therapeutic care based on a detailed understanding of the nature of combined risk between *TP53* and other variants, including CNVs.

## Conclusions

In this work we show a highly significant increase of rare CNVs in patients with mutations in the *TP53* DNA binding domain site (DBD) relative both to controls and to carriers of the mild *TP53* Brazilian variant. An accumulation of rare CNVs over generations in the presence of penetrant *TP53* mutation provides a plausible explanation for anticipation in LFS: in contrast, lack of this increase in rare CNVs combined with a longer lifespan and likelihood of reproduction probably contributes to the frequency of the p.R337H mutation being ~300 xs larger than reported for any other *TP53* mutation.

## Availability of supporting data

The data set supporting the results of this article is included within the article and its additional files.

## Abbreviations

LFS: Li-Fraumeni syndrome; LFL: Li-Fraumeni like syndrome; CNV: Copy Number Variation; DBD: DNA binding domain.

## Misc

Amanda G. Silva and Maria Isabel W Achatz are the first 2 authors that should be regarded as joint First Authors.

## Competing interests

The authors declare that they have no competing interests.

## Authors' contributions

AGS carried out the molecular genetic studies. AGS and PLP wrote the manuscript. ACVK and CR participated in the design and coordination of the study and helped writing the manuscript. MIWA recruited and selected the patients. All authors read and approved the final manuscript.

## Supplementary Material

Additional file 1 **Results of Array-CGH.** Full CNV data of LFS/LFL patients, chromosome coordinates given according to Hg18 (NCBI Build 36.1/hg18; http://genome.ucsc.edu). Click here for file

Additional file 2 **Frequency of the rare CNVs detected in the*TP53*mutated individuals.** Click here for file
