# Supplementary material for: Number of rare germline CNVs and TP53 mutation types
Source: Orphanet J Rare Dis. 2012 Dec 21;7:101. doi: 10.1186/1750-1172-7-101 (PMC3558401; doi:10.1186/1750-1172-7-101)
Supplement: Additional file 1 — Results of Array-CGH. Full CNV data of LFS/LFL patients, chromosome coordinates given according to Hg18 (NCBI Build 36.1/hg18; http://genome.ucsc.edu). [file 1750-1172-7-101-S1.docx]

**Additional File 1 - CNV Profile of all Patients -** Full CNV data of LFS/LFL patients, rare CNVs are in red. Chromosome coordinates given according to Hg18. Chr -Chromosome, Amp - Amplification.

| **Sample** | **Chr** | **Cytoband** | **Start** | **Stop** | **#Probes** | **Amp** | **Deletion** | **Gene Names** |
| --- | --- | --- | --- | --- | --- | --- | --- | --- |
| **Y0012T001** |  |  |  |  |  |  |  |  |
|  | chr5 | q12.3 | 64012317 | 64040725 | 4 | 0 | -0.876331 | FAM159B |
|  | chr2 | p22.2 | 37813011 | 37847719 | 5 | 0.828931 | 0 |  |
|  | chr3 | q26.1 | 163997028 | 164077488 | 5 | 0.903846 | 0 |  |
|  | chr4 | q28.3 | 135162486 | 135399760 | 8 | 0 | -0.947525 | PABPC4L |
|  | chr8 | p11.23 | 39356395 | 39494087 | 12 | 0.801468 | -0.801468 | ADAM5P, ADAM3A |
|  | chr16 | p13.3 | 686618 | 738487 | 6 | 0.83933 | -0.83933 | FBXL16, METRN, FAM173A, CCDC78, HAGHL, HAGHL, NARFL |
|  |  |  |  |  |  |  |  |  |
| **Y0015T000** |  |  |  |  |  |  |  |  |
|  | chr1 | q21.1 | 147308357 | 147476054 | 13 | 0 | -1.637053 |  |
|  | chr8 | p11.23 | 39356395 | 39494087 | 12 | 2.455864 | 0 | ADAM5P, ADAM3A |
|  | chr14 | q32.33 | 105962105 | 106002379 | 5 | 0 | -1.701925 |  |
|  | chr17 | q21.31 | 41598811 | 41700962 | 6 | 1.249723 | 0 | KIAA1267 |
|  | chr2 | p23.1 | 30667988 | 30701994 | 5 | 0 | -0.957854 | LCLAT1, LCLAT1 |
|  | chr2 | p22.3 | 34556234 | 34580733 | 3 | 0 | -1.001776 |  |
|  | chr4 | q13.2 | 70190482 | 70294512 | 3 | 0 | -1.326518 | UGT2B28 |
|  | chr10 | q11.22 | 46395963 | 47172734 | 24 | 0.425589 | 0 | GPRIN2, PPYR1, LOC728643, ANXA8, ANXA8L1, FAM25C, FAM25G, FAM25B, LOC642826, ANTXRL |
|  | chr15 | q11.2 | 20316792 | 20742644 | 34 | 0 | -0.781508 | TUBGCP5, TUBGCP5, CYFIP1, CYFIP1, NIPA2, NIPA1, NIPA1, WHAMML1 |
|  | chr15 | q21.3 | 53140467 | 53231823 | 3 | 0 | -1.074009 |  |
|  |  |  |  |  |  |  |  |  |
| **Y0027T000** |  |  |  |  |  |  |  |  |
|  | chr2 | p22.3 | 34556234 | 34580733 | 3 | 4.208998 | 0 |  |
|  | chr22 | q11.23 | 22677759 | 22720395 | 5 | 2.541339 | 0 | LOC391322, GSTT1, GSTTP2 |
|  | chrX | p21.3 | 26674328 | 26777592 | 4 | 1.571147 | 0 |  |
|  | chr1 | p31.1 | 72541243 | 72568209 | 3 | 3.646425 | 0 |  |
|  | chr1 | q44 | 246794352 | 246875216 | 7 | 0 | -1.249313 | OR2T34, OR2T10, OR2T11, OR2T35 |
|  | chr2 | q37.3 | 242501039 | 242677269 | 15 | 0 | -0.967482 |  |
|  | chr8 | p11.23 | 39356395 | 39464777 | 10 | 0 | -3.405984 | ADAM5P, ADAM3A |
|  | chr12 | p13.31 | 9528390 | 9604833 | 7 | 1.832319 | 0 |  |
|  | chr14 | q32.33 | 105602402 | 105630289 | 3 | 0 | -2.938967 |  |
|  |  |  |  |  |  |  |  |  |
| **Y0049T001** |  |  |  |  |  |  |  |  |
|  | chr1 | q44 | 246794352 | 246852326 | 6 | 0 | -0.773215 | OR2T34, OR2T10 |
|  | chr2 | q37.3 | 242514393 | 242690178 | 15 | 0 | -0.858715 |  |
|  | chr3 | q29 | 196904149 | 196930005 | 5 | 0 | -0.840872 |  |
|  | chr4 | q13.2 | 69074940 | 69145174 | 6 | 1.477959 | 0 | UGT2B17 |
|  | chr11 | q11 | 55134286 | 55207505 | 8 | 0 | -2.388982 | OR4P4, OR4S2, OR4C6 |
|  | chr14 | q11.1 - q11.2 | 18798441 | 19484213 | 13 | 0 | -0.795414 | OR4Q3, OR4M1, OR4N2, OR4K2, OR4K5, OR4K1 |
|  | chr15 | q11.2 | 18741516 | 20060261 | 46 | 0.63983 | 0 | LOC283755, A26B1, OR4M2, OR4N4, LOC650137 |
|  | chr6 | p21.33 | 31395130 | 31456863 | 4 | 0 | -0.844991 | HLA-B |
|  | chr6 | q14.1 | 79035691 | 79080188 | 3 | 2.759722 | 0 |  |
|  | chr14 | q32.33 | 105602402 | 105630289 | 3 | 0 | -2.699282 |  |
|  | chr22 | q11.23 | 22686490 | 22720395 | 4 | 1.238854 | 0 | GSTT1 |
|  |  |  |  |  |  |  |  |  |
| **Y0099T000** |  |  |  |  |  |  |  |  |
|  | chr1 | p31.1 | 72541243 | 72568209 | 3 | 0 | -4.237285 |  |
|  | chr2 | p22.3 | 34556234 | 34580733 | 3 | 3.858554 | 0 |  |
|  | chr3 | q26.1 | 163997028 | 164101976 | 6 | 0 | -0.914951 |  |
|  | chr4 | q13.2 | 69074940 | 69145174 | 6 | 0 | -2.075479 | UGT2B17 |
|  | chr11 | p15.4 | 7773867 | 7783544 | 3 | 0 | -1.046287 | OR5P2 |
|  | chr22 | q11.23 | 23974513 | 24241792 | 21 | 0 | -0.569815 | IGLL3, LRP5L, LRP5L |
|  |  |  |  |  |  |  |  |  |
| **Y0100T000** |  |  |  |  |  |  |  |  |
|  | chr1 | q21.3 | 150822873 | 150853058 | 3 | 0.917114 | 0 | LCE3C, LCE3B |
|  | chr1 | q44 | 246794352 | 246875216 | 7 | 0 | -0.960004 | OR2T34, OR2T10, OR2T11, OR2T35 |
|  | chr2 | p22.3 | 34556234 | 34580733 | 3 | 0 | -1.317092 |  |
|  | chr10 | q11.22 | 46395963 | 46568696 | 14 | 0.581379 | 0 | GPRIN2, PPYR1, LOC728643 |
|  | chr15 | q11.2 | 18741516 | 20060261 | 46 | 1.163907 | 0 | LOC727832, GOLGA8C, LOC646214, CXADRP2, POTEB, LOC727924, OR4M2, OR4N4, LOC650137 |
|  | chr22 | q13.1 | 37688858 | 37715585 | 3 | 0 | -1.068588 | APOBEC3A, APOBEC3B |
|  |  |  |  |  |  |  |  |  |
|  |  |  |  |  |  |  |  |  |
| **Y0106T000** |  |  |  |  |  |  |  |  |
|  | chr3 | q26.1 | 163997028 | 164101976 | 6 | 0 | -5.20139 |  |
|  | chr5 | p15.33 | 811969 | 873565 | 3 | 1.088416 | 0 | ZDHHC11 |
|  | chr11 | p15.4 | 7773867 | 7783544 | 3 | 0 | -1.12064 | OR5P2 |
|  | chr14 | q11.1 - q11.2 | 18798441 | 19497223 | 14 | 0 | -0.538284 | P704P, OR4Q3, OR4M1, OR4N2, OR4K2, OR4K5, OR4K1 |
|  | chr14 | q24.3 | 73071204 | 73092218 | 3 | 1.040109 | 0 | HEATR4, ACOT1 |
|  | chr15 | q11.2 | 18741516 | 20060261 | 46 | 0 | -0.643065 | LOC727832, GOLGA8C, LOC646214, CXADRP2, POTEB, LOC727924, OR4M2, OR4N4, LOC650137 |
|  | chr22 | q11.23 | 22677759 | 22720395 | 5 | 0 | -2.252777 | LOC391322, GSTT1, GSTTP2 |
|  |  |  |  |  |  |  |  |  |
| **Y0107T000** |  |  |  |  |  |  |  |  |
|  | chr1 | p21.1 | 103908853 | 104012720 | 5 | 0 | -0.79125 | AMY2B, AMY2A, AMY1A, AMY1A, AMY1C, AMY1B |
|  | chr1 | q44 | 246794352 | 246875216 | 7 | 0.937836 | 0 | OR2T34, OR2T10, OR2T11, OR2T35 |
|  | chr7 | q36.3 | 158085329 | 158431791 | 31 | 0.314217 | 0 | NCAPG2, FAM62B, WDR60 |
|  | chr12 | p13.31 | 9528390 | 9604833 | 7 | 0 | -1.369741 |  |
|  | chr14 | q32.33 | 105602402 | 105630289 | 3 | 0 | -2.113699 |  |
|  | chr14 | q32.33 | 105856772 | 105881877 | 3 | 1.009805 | 0 |  |
|  | chr15 | q11.2 | 18741516 | 20060261 | 46 | 0.60165 | 0 | LOC283755, A26B1, OR4M2, OR4N4, LOC650137 |
|  | chr15 | q14 | 32523041 | 32578683 | 3 | 1.038462 | 0 |  |
|  | chr17 | q12 | 31474318 | 31499768 | 3 | 1.072119 | 0 |  |
|  | chr3 | q29 | 196904149 | 196943580 | 7 | 0 | -0.835848 | MUC20, MUC20 |
|  | chr22 | q11.23 | 22677759 | 22720395 | 5 | 2.959882 | 0 | GSTT1 |
|  |  |  |  |  |  |  |  |  |
| **Y0127T000** |  |  |  |  |  |  |  |  |
|  | chr3 | q26.1 | 163997028 | 164101976 | 6 | 1.017277 | 0 |  |
|  | chr8 | p11.23 | 39356395 | 39482185 | 11 | 0 | -2.643782 | ADAM5P, ADAM3A |
|  | chr14 | q32.33 | 105856772 | 105881877 | 3 | 0 | -1.090153 |  |
|  | chr15 | q11.2 | 18741516 | 20060261 | 46 | 0 | -1.16386 | LOC727832, GOLGA8C, LOC646214, CXADRP2, POTEB, LOC727924, OR4M2, OR4N4, LOC650137 |
|  | chr16 | q22.3 | 72929586 | 72964983 | 4 | 0.971394 | 0 | LOC283922 |
|  | chr22 | q11.23 | 22677759 | 22720395 | 5 | 0.742305 | 0 | LOC391322, GSTT1, GSTTP2 |
|  |  |  |  |  |  |  |  |  |
| **Y0131T000** |  |  |  |  |  |  |  |  |
|  | chr1 | q21.3 | 150822873 | 150853058 | 3 | 0 | -1.518233 | LCE3C, LCE3B |
|  | chr2 | p22.3 | 34556234 | 34580733 | 3 | 0 | -3.706211 |  |
|  | chr4 | q13.2 | 69074940 | 69145174 | 6 | 1.995726 | 0 | UGT2B17 |
|  | chr6 | q14.1 | 79035691 | 79080188 | 3 | 0 | -4.828242 |  |
|  | chr6 | q27 | 168098317 | 168268397 | 13 | 0 | -0.902197 | MLLT4, MLLT4, KIF25, FRMD1, FRMD1 |
|  | chr14 | q24.3 | 73071204 | 73092218 | 3 | 0 | -1.378931 | HEATR4, ACOT1 |
|  |  |  |  |  |  |  |  |  |
| **Y0144T000** |  |  |  |  |  |  |  |  |
|  | chr14 | q31.1 | 79501967 | 79618136 | 4 | 0 | -1.004831 |  |
|  | chrX | p21.3 | 26674328 | 26777592 | 4 | 0 | -1.26474 |  |
|  | chr1 | p31.1 | 72541243 | 72568209 | 3 | 2.449401 | 0 |  |
|  | chr1 | q21.1 - q21.2 | 147308357 | 148081969 | 17 | 0 | -0.887898 | LOC388692, FCGR1C, FCGR1C, PPIAL4C, PPIAL4A, LOC728855, FCGR1A, HIST2H2BF, HIST2H3D, HIST2H4A, HIST2H4B, HIST2H3C, HIST2H3A, HIST2H2AA4, HIST2H2AA3 |
|  | chr2 | p22.3 | 34556234 | 34580733 | 3 | 0 | -2.3539 |  |
|  | chr8 | p11.23 | 39368309 | 39482185 | 10 | 0.993772 | 0 | ADAM5P, ADAM3A |
|  | chr11 | q11 | 55134286 | 55207505 | 8 | 0.574404 | 0 | OR4P4, OR4S2, OR4C6 |
|  | chr12 | p13.31 | 9528390 | 9604833 | 7 | 0.742455 | 0 |  |
|  | chr15 | q11.2 | 18692665 | 20060261 | 47 | 1.09331 | 0 | LOC727832, GOLGA8C, LOC646214, CXADRP2, POTEB, LOC727924, OR4M2, OR4N4, LOC650137 |
|  |  |  |  |  |  |  |  |  |
| **Y0154T000** |  |  |  |  |  |  |  |  |
|  | chr14 | q24.3 | 73071204 | 73092218 | 3 | 0 | -2.4029 | HEATR4, ACOT1 |
|  | chr2 | p22.3 | 34556234 | 34580733 | 3 | 4.285968 | 0 |  |
|  | chr3 | q26.1 | 164038717 | 164101976 | 4 | 0.811893 | 0 |  |
|  | chr8 | p11.23 | 39356395 | 39464777 | 10 | 0.975725 | 0 | ADAM5P, ADAM3A |
|  | chr11 | p11.12 | 49671067 | 49710227 | 3 | 0 | -1.582584 | LOC440040 |
|  |  |  |  |  |  |  |  |  |
| **Y0001T000** |  |  |  |  |  |  |  |  |
|  | chr2 | p22.3 | 34556234 | 34580733 | 3 | 1.794837 | 0 |  |
|  | chr11 | q11 | 55134286 | 55207505 | 8 | 0.687118 | 0 | OR4P4, OR4S2, OR4C6 |
|  | chr14 | q32.33 | 105856772 | 105881877 | 3 | 1.113592 | 0 |  |
|  | chr15 | q11.2 | 19805760 | 20060261 | 22 | 0 | -0.723763 | LOC727924, OR4M2, OR4N4, LOC650137 |
|  | chr16 | p11.2 - p11.1 | 34350507 | 34584993 | 9 | 0.590533 | 0 | LOC283914, LOC283914, LOC146481 |
|  | chr17 | q21.31 | 41527505 | 41700962 | 12 | 0.432705 | 0 | KIAA1267 |
|  | chr22 | q11.23 | 22677759 | 22720395 | 5 | 1.036988 | 0 | LOC391322, GSTT1, GSTTP2 |
|  |  |  |  |  |  |  |  |  |
| **Y0033T000** |  |  |  |  |  |  |  |  |
|  | chr1 | p31.1 | 72541243 | 72568209 | 3 | 0 | -2.301302 |  |
|  | chr8 | p11.23 | 39356395 | 39482185 | 11 | 1.379622 | 0 | ADAM5P, ADAM3A |
|  | chr10 | q26.3 | 135084577 | 135254661 | 13 | 0.772902 | 0 | SPRN, LOC619207, CYP2E1, SYCE1, SYCE1, SYCE1 |
|  | chr12 | p12.3 | 16355438 | 16502869 | 10 | 0 | -0.928156 | MGST1, MGST1, MGST1, MGST1 |
|  | chr14 | q32.33 | 105602402 | 105630289 | 3 | 0 | -2.141083 |  |
|  | chr14 | q32.33 | 106222937 | 106251281 | 3 | 1.408912 | 0 |  |
|  | chr14 | q11.1 - q11.2 | 18798441 | 19484213 | 13 | 0.732525 |  | P704P, OR4Q3, OR4M1, OR4N2, OR4K2, OR4K5, OR4K1 |
|  | chr16 | q22.1 | 68710077 | 68751590 | 3 | 0.98497 |  | PDPR |
|  | chr20 | p13 | 1516766 | 1547058 | 3 | 1.024432 |  | SIRPB1, SIRPB1 |
|  | chr22 | q11.23 | 22677759 | 22720395 | 5 | 0.98071 |  | LOC391322, GSTT1, GSTTP2 |
|  |  |  |  |  |  |  |  |  |
| **Y0053T000** |  |  |  |  |  |  |  |  |
|  | chr11 | q11 | 55134286 | 55207505 | 8 | 0 | -0.790167 | OR4P4, OR4S2, OR4C6 |
|  | chr14 | q11.1 - q11.2 | 18798441 | 19484213 | 13 | 0.464901 | 0 | OR4Q3, OR4M1, OR4N2, OR4K2, OR4K5, OR4K1 |
|  | chr16 | p11.2 | 32481109 | 33559407 | 19 | 0 | -0.525726 | LOC729355, TP53TG3, LOC729355, TP53TG3, LOC729355, TP53TG3 |
|  | chr22 | q11.23 | 22617849 | 22667808 | 4 | 0 | -0.843903 | GSTT2B, GSTT2, DDTL, DDT, DDT, GSTT2 |
|  | chr22 | q11.23 | 22677759 | 22720395 | 5 | 1.928584 | 0 | GSTT1 |
|  |  |  |  |  |  |  |  |  |
| **Y0057T000** |  |  |  |  |  |  |  |  |
|  | chr1 | q21.3 | 150822873 | 150853058 | 3 | 1.685302 | 0 | LCE3C, LCE3B |
|  | chr2 | q13 | 109784284 | 110337831 | 14 | 0.4776 | 0 | RGPD5, RGPD7, RGPD6, RGPD5, LIMS3, LIMS3-LOC440895, LIMS3-LOC440895, LOC440895, MALL, NPHP1, NCRNA00116 |
|  | chr7 | p21.2 | 14096856 | 14182992 | 7 | 0 | -0.966597 | DGKB, DGKB |
|  | chr16 | q22.3 | 71056472 | 71272532 | 9 | 0 | -0.793426 |  |
|  | chr22 | q11.23 | 22686490 | 22720395 | 4 | 0 | -3.06969 | LOC391322, GSTT1, GSTTP2 |
|  | chrX | q28 | 154049985 | 154079019 | 3 | 0 | -1.034514 |  |
|  |  |  |  |  |  |  |  |  |
| **Y0065T000** |  |  |  |  |  |  |  |  |
|  | chr1 | q21.3 | 150822873 | 150853058 | 3 | 0.869717 | 0 | LCE3C, LCE3B |
|  | chr6 | q14.1 | 79035691 | 79080188 | 3 | 1.004531 | 0 |  |
|  | chrX | q28 | 154049985 | 154079019 | 3 | 0 | -1.101854 |  |
|  | chr2 | q33.1 | 201871706 | 201930393 | 5 | 0 | -0.947716 | ALS2CR12 |
|  | chr5 | q21.1 | 99352396 | 99447696 | 7 | 0 | -0.729398 |  |
|  | chr8 | p23.1 | 7156700 | 7824259 | 9 | 0 | -1.255604 | DEFB109, DEFB103A, DEFB103B, SPAG11B, SPAG11B, SPAG11B, SPAG11B, DEFB104A, DEFB104B, DEFB106B, DEFB106A, DEFB105B, DEFB105A, DEFB107A, DEFB107B, FAM90A7, FAM90A7, DEFB107B, DEFB107A, DEFB105B, DEFB105A, DEFB106A, DEFB106B, DEFB104A, DEFB104B, SPAG11B, SPAG11A, DEFB103B, DEFB103A, DEFB4 |
|  | chr8 | p11.23 | 39356395 | 39494087 | 12 | 0 | -3.070266 | ADAM5P, ADAM3A |
|  | chr10 | q26.3 | 135103829 | 135227663 | 11 | 0.568641 | 0 | LOC619207, CYP2E1, SYCE1, SYCE1, SYCE1 |
|  | chr14 | q11.1 - q11.2 | 18798441 | 19484213 | 13 | 0.613316 | 0 | P704P, OR4Q3, OR4M1, OR4N2, OR4K2, OR4K5, OR4K1 |
|  | chr15 | q11.2 | 18692665 | 19382727 | 22 | 0 | -0.423067 | LOC727832, GOLGA8C, LOC646214, CXADRP2, POTEB |
|  | chr22 | q11.23 | 22677759 | 22720395 | 5 | 0 | -3.534804 | LOC391322, GSTT1, GSTTP2 |
|  |  |  |  |  |  |  |  |  |
| **Y0079T000** |  |  |  |  |  |  |  |  |
|  | chr12 | p13.31 | 9528390 | 9604833 | 7 | 0.854949 | 0 |  |
|  | chr17 | q21.31 | 41527505 | 41700962 | 12 | 0 | -0.432705 | KIAA1267 |
|  | chr1 | q44 | 246794352 | 246875216 | 7 | 0.748934 | 0 | OR2T34, OR2T10, OR2T11, OR2T35 |
|  | chr2 | p22.3 | 34556234 | 34580733 | 3 | 0 | -4.222684 |  |
|  | chr3 | q26.1 | 164038717 | 164101976 | 4 | 1.034853 | 0 |  |
|  |  |  |  |  |  |  |  |  |
| **Y0087T000** |  |  |  |  |  |  |  |  |
|  | chr1 | q32.2 | 208179362 | 208208583 | 3 | 0 | -1.401819 | SYT14, SYT14, SYT14 |
|  | chr2 | p22.3 | 34556234 | 34580733 | 3 | 0 | -2.241951 |  |
|  | chr3 | q26.1 | 163997028 | 164101976 | 6 | 1.869373 | 0 |  |
|  | chr5 | q21.3 | 107277661 | 107309812 | 3 | 0 | -1.114786 | FBXL17 |
|  | chr7 | q34 | 142507901 | 142600931 | 9 | 0 | -0.718519 | PIP, TAS2R39 |
|  | chr8 | p11.23 | 39356395 | 39482185 | 11 | 0.713066 | 0 | ADAM5P, ADAM3A |
|  | chr11 | p15.4 | 4927845 | 4967630 | 4 | 0 | -0.923494 | OR51A2, MMP26 |
|  | chr13 | q14.2 | 46931931 | 47023874 | 4 | 0 | -0.830971 |  |
|  | chr14 | q11.2 | 21688496 | 22006035 | 30 | 0.358974 | 0 |  |
|  | chr15 | q13.1 | 27759318 | 27780226 | 3 | 0 | -1.144307 | TJP1 |
|  |  |  |  |  |  |  |  |  |
| **Y0097T001** |  |  |  |  |  |  |  |  |
|  | chr1 | p31.1 | 72541243 | 72568209 | 3 | 4.878582 | 0 |  |
|  | chr8 | p23.1 | 7156700 | 7824259 | 9 | 1.475967 | 0 | DEFB109, DEFB103A, DEFB103B, SPAG11B, SPAG11B, SPAG11B, SPAG11B, DEFB104A, DEFB104B, DEFB106B, DEFB106A, DEFB105B, DEFB105A, DEFB107A, DEFB107B, FAM90A7, FAM90A7, DEFB107B, DEFB107A, DEFB105B, DEFB105A, DEFB106A, DEFB106B, DEFB104A, DEFB104B, SPAG11B, SPAG11A, DEFB103B, DEFB103A, DEFB4 |
|  | chr14 | q11.2 | 19273089 | 19484213 | 12 | 0.63366 | 0 | OR4Q3, OR4M1, OR4N2, OR4K2, OR4K5, OR4K1 |
|  | chr14 | q32.33 | 105602402 | 105630289 | 3 | 1.081365 | 0 |  |
|  |  |  |  |  |  |  |  |  |
| **Y0103T002** |  |  |  |  |  |  |  |  |
|  | chrX | q28 | 148653235 | 148789920 | 8 | 0.62793 | 0 | HSFX1, HSFX1, MAGEA9B, MAGEA9, MAGEA8 |
|  | chr1 | p21.1 | 103908853 | 104012720 | 5 | 0.811739 | 0 | AMY2B, LOC648740, AMY2A, AMY1A, AMY1A, AMY1C, AMY1B |
|  | chr1 | q44 | 246794352 | 246875216 | 7 | 0 | -0.917346 | OR2T34, OR2T10, OR2T11, OR2T35 |
|  | chr2 | p22.3 | 34556234 | 34580733 | 3 | 0 | -4.258987 |  |
|  | chr5 | p15.2 | 12645163 | 12722931 | 4 | 0 | -0.855524 | TAG |
|  | chr6 | q14.1 | 79035691 | 79080188 | 3 | 0 | -0.948001 |  |
|  | chr7 | q21.13 | 89629989 | 90167047 | 41 | 0.520313 | 0 | STEAP1, STEAP2, STEAP2, STEAP2, C7orf63, GTPBP10, CLDN12 |
|  | chr15 | q14 | 32523041 | 32578683 | 3 | 0 | -1.017972 |  |
