# Supplementary material for: Number of rare germline CNVs and TP53 mutation types
Source: Orphanet J Rare Dis. 2012 Dec 21;7:101. doi: 10.1186/1750-1172-7-101 (PMC3558401; doi:10.1186/1750-1172-7-101)
Supplement: Additional file 2 — Frequency of the rare CNVs detected in theTP53mutated individuals. [file 1750-1172-7-101-S2.docx]

Additional File 2 – Frequency of the rare CNVs detected in the *TP53* mutated individuals.

| Chrom | start | end | CNV type | genes | DGV  (n=8058) | dbVAR [1] (n=8148) | Frequency of CNVs (%)  (DGV + dbVAR) |
| --- | --- | --- | --- | --- | --- | --- | --- |
| chr5 | 64012317 | 64040725 | del | *FAM159B* | 1/776 [2] | 8/8148 | 0.05 |
| chr4 | 135162486 | 135399760 | del | *PABPC4L* | 3 /1190 [3] | 10/8148 | 0.08 |
| chr12 | 16355438 | 16502869 | del | *MGST1* | 0 | 4/8148 | 0.02 |
| chr7 | 14096856 | 14182992 | del | *DGKB* | 2/20 [4] | 0 | 0.01 |
| chr2 | 201871706 | 201930393 | del | *ALS2CR12* | 0 | 1/8148 | 0.01 |
| chr1 | 208179362 | 208208583 | del | *SYT14* | 0 | 1/8148 | 0.01 |
| chr11 | 4927845 | 4967630 | del | *MMP26* | 0 | 1/8148 | 0.01 |
| chr15 | 27759318 | 27780226 | del | *TJP1* | 1/776 [2] | 0 | 0.01 |
| chr5 | 12645163 | 12722931 | del | *TAG* | 0 | 11/8148 | 0.07 |
| chr7 | 158085329 | 158431791 | dup | *NCAPG2, ESYT2, WRD60* | 0 | 7/8148 | 0.04 |
| chr2 | 109784284 | 110337831 | dup | *RGPD5, RGPD8, RGPD6, LIMS3L* | 1/776 [2] | 8/8148 | 0.06 |
| chrX | 148653235 | 148789920 | dup | *HSFX1, HSFX2, MAGEA9, MAGEA9B* | 1/112 [5] | 0 | 0.01 |
| chr7 | 89629989 | 90167047 | dup | *C7orf63, GTPBP10, CLDN12* | 1/39 [6] | 1/8148 | 0.01 |

References

1. Xu H, Poh WT, Sim X, Ong RT, Suo C, Tay WT, Khor CC, Seielstad M, Liu J, Aung T *et al*: **SgD-CNV, a database for common and rare copy number variants in three Asian populations**. *Hum Mutat* 2011, **32**(12):1341-1349.

2. Pinto D, Marshall C, Feuk L, Scherer SW: **Copy-number variation in control population cohorts**. *Hum Mol Genet* 2007, **16 Spec No. 2**:R168-173.

3. Zogopoulos G, Ha KC, Naqib F, Moore S, Kim H, Montpetit A, Robidoux F, Laflamme P, Cotterchio M, Greenwood C *et al*: **Germ-line DNA copy number variation frequencies in a large North American population**. *Hum Genet* 2007, **122**(3-4):345-353.

4. Sebat J, Lakshmi B, Troge J, Alexander J, Young J, Lundin P, Månér S, Massa H, Walker M, Chi M *et al*: **Large-scale copy number polymorphism in the human genome**. *Science* 2004, **305**(5683):525-528.

5. Wang K, Li M, Hadley D, Liu R, Glessner J, Grant SF, Hakonarson H, Bucan M: **PennCNV: an integrated hidden Markov model designed for high-resolution copy number variation detection in whole-genome SNP genotyping data**. *Genome Res* 2007, **17**(11):1665-1674.

6. Conrad DF, Pinto D, Redon R, Feuk L, Gokcumen O, Zhang Y, Aerts J, Andrews TD, Barnes C, Campbell P *et al*: **Origins and functional impact of copy number variation in the human genome**. *Nature* 2010, **464**(7289):704-712.
